# Factors associated with maternal well-being during childbirth among postpartum women in Minas Gerais

**DOI:** 10.1590/0034-7167-2023-0304

**Published:** 2024-12-16

**Authors:** Talyta Sâmara Batista Ferreira, Cinara Botelho Moutinho, Edmar Rocha Almeida, Ana Júlia Soares Oliveira, Carolina Amaral Oliveira Rodrigues, Clara de Cássia Versiani, Sibylle Emilie Vogt, Marise Fagundes Silveira

**Affiliations:** I Universidade Estadual de Montes Claros. Montes Claros, Minas Gerais, Brazil; II Hospital Santo Antônio. Taiobeiras, Minas Gerais, Brazil; III Secretaria Municipal de Saúde de Taiobeiras. Taiobeiras, Minas Gerais, Brazil

**Keywords:** Maternal Welfare, Postpartum Period, Delivery Obstetric, Midwifery, Humanization of Assistance., Bienestar Materno, Periodo Posparto, Parto Obstétrico, Partería, Humanización de la Atención.

## Abstract

**Objectives:**

to analyze the factors associated with maternal well-being during childbirth among postpartum women in Minas Gerais.

**Methods:**

a cross-sectional study nested within a cohort was conducted with postpartum women in a municipality of Minas Gerais. The Maternal Well-being in Childbirth Scale 2 was used. The prevalence of maternal well-being during childbirth was estimated. The magnitude of the association between maternal distress and care practices was estimated using the Prevalence Ratio (PR), applying Poisson regression.

**Results:**

a total of 183 postpartum women aged between 15 and 46 years participated, with 26.2%, 27.9%, and 45.9% reporting excellent, adequate, and poor well-being during childbirth care, respectively. Maternal distress was more prevalent among women who underwent cesarean sections (PR = 1.60) and those who did not receive breastfeeding information (PR = 1.59).

**Conclusions:**

a high prevalence of maternal distress during childbirth was observed, associated with cesarean delivery and the lack of breastfeeding information.

## RESUMEN

**Objetivos:**

analizar los factores asociados al bienestar materno en situación de parto de puérperas en Minas Gerais.

**Métodos:**

estudio transversal, anidado en una cohorte, realizado con puérperas en un municipio de Minas Gerais. Se adoptó la escala de Bienestar Materno en Situación de Parto 2. Se estimaron las prevalencias de bienestar materno en situación de parto. La magnitud de la asociación entre malestar materno y prácticas asistenciales fue estimada por la Razón de Prevalencia (RP), utilizando la regresión de Poisson.

**Resultados:**

participaron 183 puérperas con edades entre 15 y 46 años, de las cuales el 26,2%, 27,9% y 45,9% reportaron, respectivamente, excelente, adecuado y malestar en la asistencia al parto. El malestar materno fue más prevalente entre puérperas que tuvieron un parto por cesárea (RP = 1,60) y que no recibieron información sobre lactancia (RP = 1,59).

**Conclusiones:**

se observó una alta prevalencia de malestar durante el parto, asociada a la realización de cesáreas y a la falta de información sobre lactancia.

## INTRODUCTION

Since the 1990s, significant advancements have been made in public policies for maternal and child health in Brazil, with a focus on humanizing care. These advancements aim to improve prenatal, childbirth, and postpartum care, ensure access and support for pregnant women, and reduce infant and maternal mortality. The desired care model is based on pillars such as respecting women’s needs and the physiology of labor and childbirth, providing objective information, fostering empathetic and non-authoritarian interpersonal relationships between the woman in labor and the healthcare professional, including women in decision-making, and promoting teamwork that recognizes and values obstetric nurses and midwives, as well as the use of evidence-based care practices and interventions^(1,2)^.

Nevertheless, inappropriate practices remain common in obstetric and neonatal care. Women and newborns are often subjected to unnecessary interventions, such as the routine use of oxytocin, episiotomy, cesarean section, and nasopharyngeal aspiration of the newborn, among others. Additionally, reports of disrespectful behavior by professionals during interactions with women in labor are frequent^(1,2)^.

Data published by the World Health Organization (WHO) show that between 2010 and 2018, cesarean deliveries accounted for 21.1% of births worldwide, 42.8% in Latin America and the Caribbean, and 55.7% in Brazil, which ranks second globally^(3)^. In 2019, the proportion of cesarean deliveries was 58.08% in Minas Gerais and 49.71% in the city of Montes Claros, the study setting^(4)^. The city has three hospitals that provide childbirth care services, predominantly publicly funded, with two of them serving as training centers for obstetricians and one for obstetric nurses.

Beyond ensuring a safe birth with evidence-based practices, it is essential to provide a positive experience for women in llabor and their families^(1,5)^. Maternal well-being during childbirth and satisfaction with the experience refer to the woman’s perception of the attitudes, behaviors, and care practices of the professionals. Satisfaction with the childbirth experience is also influenced by the fulfillment of expectations, the level of information provided to women, and the outcome of the birth-whether it resulted in a healthy newborn and the absence of maternal or neonatal complications^(6)^. Feeling welcomed and understood by professionals, with respect for privacy and the ability to exercise autonomy in the care process, along with a quality infrastructure, are key factors for women and contribute to well-being and satisfaction^(7)^.

Pregnancy and childbirth are unique experiences, marked by strong emotions, that place women in situations of extreme vulnerability. Negative experiences, abuse, and obstetric violence during care are associated with depressive disorders, which negatively impact women’s quality of life and their ability to care for themselves and their newborns^(8,9)^. While childbirth experiences are inherently subjective, they can be objectively measured, which has been the focus of numerous evaluation proposals ^(10)^. One of the instruments validated for the Brazilian cultural context is the Maternal Well-being in Childbirth Scale 2 (BMSP2)^(11)^, which has been used in national studies to estimate the prevalence of well-being during childbirth^(12,13)^.

It is important to evaluate maternal well-being as a marker of care quality. Few studies investigate this outcome, with qualitative studies predominating. Furthermore, most quantitative research does not utilize validated instruments to assess well-being during and after childbirth^(10)^.

## OBJECTIVES

To analyze the factors associated with maternal well-being during childbirth among postpartum women in Minas Gerais.

## METHODS

### Ethical aspects

The study was conducted in accordance with national and international ethical guidelines and was approved by the Research Ethics Committee of the State University of Montes Claros, whose approvals are attached to this submission. Informed consent was obtained from all participants aged over 18 years through a signed form. Participants aged 18 years or younger, as well as their legal guardians, signed the Assent Form and the Informed Consent Form.

### Study design, location, and period

This is a cross-sectional and analytical epidemiological study nested within the “ALGE Study - Assessment of the Health Conditions of Pregnant Women in Montes Claros-MG: A Longitudinal Study”, conducted in the urban area of Montes Claros, MG, Brazil, between 2018 and 2020. The ALGE Study aimed to assess the health conditions of pregnant women, postpartum women, and children served by the Family Health Strategy (FHS) services in Montes Claros.

### Population or sample, inclusion and exclusion criteria

The ALGE Study was conducted in three phases. The population in the first phase (baseline) consisted of all pregnant women (N=1661) registered in the FHS between 2018 and 2019. Of these, those who were in their first trimester of pregnancy (N=448) were invited to participate in the second phase, which took place during the third trimester of pregnancy, and in the third phase, which occurred 40 to 70 days postpartum.

The sample size for the baseline (first phase) was determined to estimate population parameters with a prevalence of 50% and a confidence interval of 95% (precision level of 2.0%). A correction was made for a finite population (N=1,661 pregnant women), with an additional 20% to account for possible non-responses and losses. The calculations indicated the need for at least 1,180 participants.

The present study refers to the third phase of this cohort, whose population consisted of 448 postpartum women registered with FHS teams in the urban area of Montes Claros, who were in their first trimester of pregnancy during the first phase of the ALGE Study. Postpartum women whose deliveries occurred during the study period were eligible. Exclusion criteria included postpartum women who had a multiple pregnancy and those who did not receive immediate assistance during childbirth (e.g., home births).

For the sample size calculation for the third phase of the ALGE Study, the formula for cross-sectional studies was used, with a correction for a finite population (N=448). The following parameters were applied: an estimated population proportion of 50%, a margin of error of 5%, a confidence level of 95%, and an additional 10% to account for non-responses and losses, resulting in a sample size of 229 postpartum women.

There was no random selection of the sample; all pregnant women registered in the FHS between 2018 and 2019 were invited to participate in the study.

### Study Protocol

In 2019, data were collected through in-person interviews conducted at the women’s homes at pre-scheduled times. In 2020, due to the COVID-19 pandemic, data were collected via an online form (Google Forms), with the link sent to the postpartum women’s email or WhatsApp. The interview team, composed of healthcare professionals and academics involved in scientific research, received training to standardize the procedure.

The following variables were analyzed: (1) sociodemographic characteristics of the postpartum women (age, education level, marital status, and source of payment for childbirth); (2) childbirth-related care variables (attending healthcare professional, type of delivery, presence of a companion, position during childbirth, use of the Kristeller maneuver, repetitive or multiple-person vaginal examinations, parallel conversations among professionals on unrelated topics during childbirth, destination of the newborn after birth, skin-to-skin contact with the newborn, breastfeeding within the first hour, receipt of breastfeeding guidance, and breastfeeding support); and (3) maternal well-being during childbirth.

To evaluate maternal well-being during childbirth, the Maternal Well-being in Childbirth Scale 2 (BMSP2), culturally adapted and validated for Brazilian Portuguese^(11)^, was used. This instrument consists of 47 items with Likert scale response options ranging from one (strongly disagree) to five (strongly agree). The items are distributed across seven domains: quality of the caregiver-patient relationship (13 items), self-care and comfort (9 items), conditions facilitating mother-child contact (4 items), depersonalized care (6 items), continuous family participation (4 items), timely and respectful care (6 items), and a comfortable physical environment (5 items). The total scale score defines three levels of maternal well-being: excellent (score > 200), adequate (score > 183 and < 200), and poor (score < 183)^(11)^.

### Analysis of Results and Statistics

Categorical variables were described using their absolute and relative frequency distributions. The scores for the BMSP2 scale domains were obtained by summing the responses to the respective items, and the total scale score was obtained by summing the responses to all items. It is important to note that the scores for items 5, 32, 33, 35, and 43, which correspond to the “depersonalized care” domain, were inverted before summation.

Descriptive measures (mean, standard deviation, maximum, and minimum values) were calculated for the total score and the seven domains of the scale, as well as the cutoff values that corresponded to 77.8% of the maximum possible score for each domain and the total score^(12)^. This percentile (77.8%) was defined in a study on the psychometric properties of the BMSP2 scale^(12)^. Additionally, the proportions of women who scored above the respective cutoff points in the domains and the total scale were estimated, indicating a positive experience with childbirth care ^(12)^. Prevalence rates, with 95% confidence intervals, were estimated for the three categories of maternal well-being during childbirth (excellent, adequate, and poor), and a histogram and column chart were created to present the obtained scores.

To assess the association between maternal well-being during childbirth (dependent variable) and sociodemographic characteristics, gestational age, and childbirth-related care variables (independent variables), the categories of the dependent variable “excellent” and “adequate” were grouped, as they represent positive outcomes. A bivariate analysis was conducted using the Chi-square or Fisher’s Exact test. Independent variables that showed a significant association with maternal well-being during childbirth at a significance level of 0.25 were selected for the multiple analysis.

In the multiple analysis, the Poisson regression model with robust variance was adopted. Prevalence ratios (PR) with 95% confidence intervals were estimated. The backward method was used to enter variables into the multiple model. For model adjustment, variables that presented a p-value ≤ 0.10 were retained in the final model. A significance level of 0.05 was adopted to consider the association with the dependent variable as significant. The Deviance Test was used to assess the goodness of fit of the final model. Data were analyzed using IBM SPSS Statistics version 23.0 for Windows^®^.

## RESULTS

A total of 183 postpartum women participated in the study, with ages ranging from 15 to 46 years and an average age of 26.3 years. The average postpartum period at the time of the interview was 59.6 days (standard deviation = 12.4 days). The majority (49.2%) were aged between 20 and 29 years, and more than two-thirds (67.6%) had incomplete or complete secondary education. The other sociodemographic characteristics and childbirth-related care details of the postpartum women are described in Table 1.

**Table 1:** Distribution of postpartum women according to sociodemographic variables and childbirth care, Montes Claros, Minas Gerais, Brazil, 2019-2020

Variable	n^*^	%
Sociodemographic variables		
Age group (years)		
15 to 19 years	29	15.8
20 to 29 years	90	49.2
30 years or older	64	35.0
Education		
Incomplete or complete elementary	23	12.6
Incomplete or complete secondary	123	67.6
Incomplete or complete higher education	36	19.8
Marital status		
Married or consensual union	139	75.9
Single/Separated/Divorced/Widowed	44	24.1
Source of payment for childbirth		
Public (SUS)	164	89.6
Private (insurance or out-of-pocket)	19	10.4
Childbirth care variables		
Type of delivery		
Vaginal	102	55.7
Cesarean	81	44.3
Attending professional		
Obstetrician	164	**92.1**
Obstetric nurse	14	**7.9**
Presence of companion	174	95.1
Horizontal position during childbirth^1^	88	86.3
Kristeller maneuver	10	5.5
Repetitive vaginal exams	30	16.4
Vaginal exams performed by different individuals	19	10.4
Parallel conversations among professionals	31	16.9
Newborn stayed with the mother after birth	156	85.2
Skin-to-skin contact with the newborn after birth	166	90.7
Breastfeeding within the first hour	142	77.6
Received breastfeeding guidance	162	88.5
Received breastfeeding support	150	88.5
Total	183	100.0

* *Totals vary due to missing data; 1Sample of women who had a vaginal delivery.*

**Figure 1:**
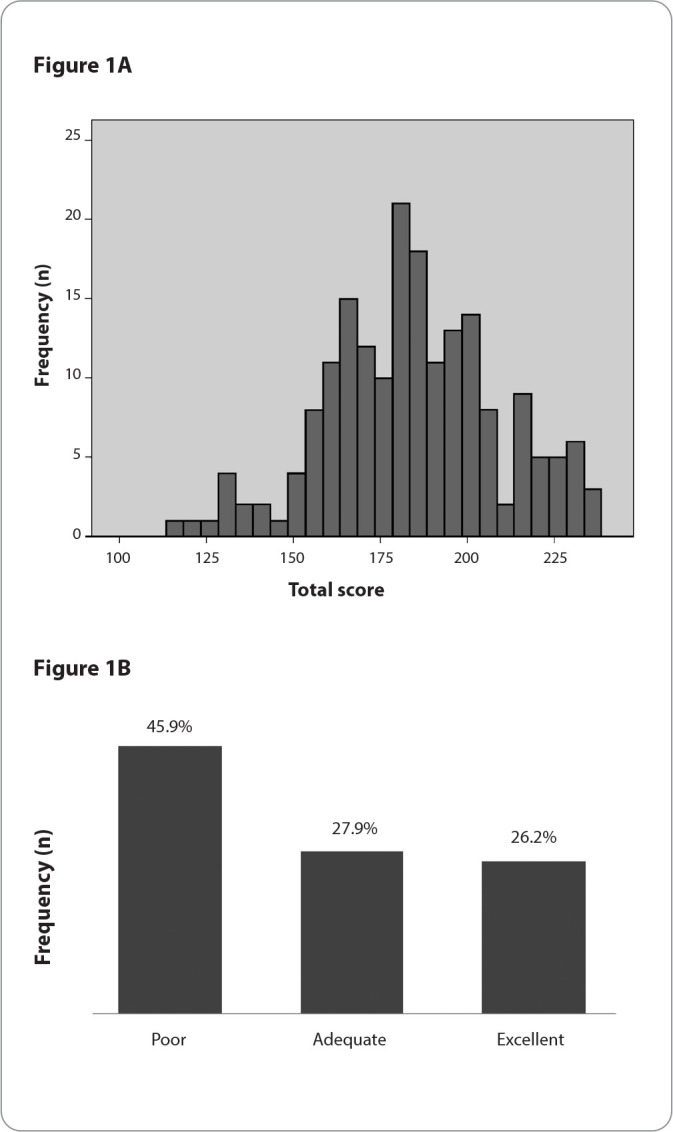
Distribution of the total score on the Maternal Well-being in Childbirth Scale (Figure 1A) and classification of postpartum women according to the scale score (Figure 1B), Montes Claros, Minas Gerais, Brazil, 2019-2020

Regarding maternal well-being during childbirth, Table 2 presents the values obtained for the domains and the total score of the BMSP2 scale. The higher the score, the more positive the evaluation of well-being. The total scale scores ranged from 116.0 to 235.0, with a mean of 184.7 and 25th and 75th percentiles of 168.0 and 201.0, respectively (Figure 1A). With the exception of the “Self-care and Comfort” and “Depersonalized Care” domains, all other domains had mean values above the cutoff point, which corresponded to 77.8% of the maximum possible score. Regarding the total score, 54.1% of the women had a positive experience (adequate or excellent) with childbirth care, meaning they had scores above the cutoff point. The proportion of the three categories of well-being according to the BMSP2 scale is presented in Figure 1B.

**Table 2:** Descriptive measures of the Maternal Well-being in Childbirth Scale 2 and classification of postpartum women according to scale scores, Montes Claros, Minas Gerais, Brazil, 2019-2020

Domains of the BMSP 2 Scale	No. of items	Possible range	Obtained range	Mean (SD)	cutoff point^*^ 77.8%	% above the cutoff point^**^
I - Quality of the caregiver-patient relationship	13	13 - 65	28 - 65	54.4 (8.4)	50.6	80.3
II - Self-care and comfort	9	9 - 45	9 - 45	28.4 (8.8)	35.0	21.3
III - Conditions that facilitate mother-child contact	4	4 - 20	4 - 20	16.5 (3.0)	15.6	74.9
IV - Depersonalized care	6	6 - 30	8 - 30	22.5 (4.1)	23.4	45.9
V - Continuous family participation	4	4 - 20	11 - 20	17.3 (2.1)	15.6	85.2
VI - Timely and respectful care	6	6 - 30	13 - 30	24.6 (3.5)	23.4	67.8
VII - Comfortable physical environment	5	5 - 25	11 - 25	20.1 (3.0)	19.5	75.4
Total	47	47 - 235	116 - 235	184.7 (25.0)	183.0	54.1
**Classification of BMSP 2**	**Score**			**n**	**% [95%CI]**	
Poor	< 183			84	45,9 [38,5 - 52,9]	
Adequate	183 ≥ x ≥ 200			51	27,9 [22,0 - 35,9]	
Excellent	> 200			48	26,2 [20,5 - 33,2]	
Total	-			183	100,0	

BMSP - Maternal well-being during childbirth; SD: standard deviation;

* Cutoff point - 77.8% of the maximum possible value;

** Percentage of women with scores above the cutoff point; CI - confidence interval.

The following childbirth-related care variables showed a significant association, at the 0.25 level, with maternal well-being during childbirth: attending professional, type of delivery, parallel conversations among professionals about other topics during childbirth, and receipt of breastfeeding guidance. These variables were selected for multiple analysis (Table 3).

**Table 3:** Association between Maternal Well-being in Childbirth and sociodemographic and childbirth care variables, Montes Claros, Minas Gerais, Brazil, 2019-2020

Variable	Classification of the BMSP2 scale		*p* value^*^
	Adequate/Excellent	Poor	
	n (%)	n (%)	
Sociodemographic Variables			
Maternal age			0.956
15 to 19 years	16 (56.7)	13(43.3)	
20 to 29 years	49 (53.9)	41(46.1)	
30 years or older	34 (53.4)	30 (46.1)	
Education			0.444
Incomplete or complete elementary	10 (42.9)	13 (57.1)	
Incomplete or complete secondary	66 (53.7)	57 (46.3)	
Incomplete or complete higher education	22 (60.6)	14 (46.3)	
Marital status			0.446
Married/Consensual union	73 (52.5)	66 (47.5)	
Single/Separated/Divorced/Widowed	26 (59.1)	18 (40.9)	
Source of payment for childbirth			0.892
Public (SUS)	89 (54.3)	75 (45.7)	
Private (insurance or out-of-pocket)	10 (52.6)	9 (47.4)	
Childbirth care variables			
Attending professional			0.013
Obstetrician	84 (51.2)	80 (48.8)	
Obstetric nurse	12 (85.7)	2 (14.3)	
Type of delivery			**<0.001**
Vaginal	68 (66.7)	34 (33.3)	
Cesarean	31 (38.3)	50 (61.7)	
Presence of companion	95 (54.6)	79 (45.4)	0.551
Horizontal birthing position^1^	59 (65.9)	29 (34.1)	0.861
Kristeller maneuver	7 (70.0)	3 (30.0)	0.299
Repetitive vaginal exams	14 (46.7)	16 (53.3)	0.372
Vaginal exams performed by different individuals	9 (47.4)	10 (52.6)	0.534
Parallel conversations among professionals	12 (38.7)	19 (61.3)	0.059
Newborn stayed with the mother after birth	86 (55.1)	70 (44.9)	0.401
Skin-to-skin contact with the newborn after birth	91 (54.8)	75 (45.2)	0.541
Breastfeeding within the first hour	78 (54.9)	64 (45.1)	0.675
Received breastfeeding guidance	93 (57.4)	69 (42.6)	0.013
Received breastfeeding support	83 (55.3)	67 (44.7)	0.475
Total	99 (54.1)	84 (45.9)	

BEMSP - Maternal well-being during childbirth;

* Chi-square test; 1Sample of women who had a vaginal delivery.

Table 4 presents the results of the multiple analysis. It was observed that the prevalence of distress during childbirth was 60.0% higher among women who had a cesarean delivery compared to those who had a vaginal delivery (PR=1.60). Among women who did not receive breastfeeding guidance, the prevalence of distress during childbirth was 59.0% higher than among those who received guidance (PR=1.59). The other variables analyzed did not show a significant association with distress during childbirth after model adjustment. The final model demonstrated good fit (Deviance test p-value = 0.897).

**Table 4:** Adjusted Poisson regression model of childbirth care variables associated with distress during childbirth, Montes Claros, Minas Gerais, Brazil, 2019-2020

Variable	PR [95% CI]	*p* value^*^
Type of delivery		
Vaginal	1.00	
Cesarean	1.60 [1.17-2.21]	0.004
Received breastfeeding guidance		
Yes	1.00	
No	1.59 [1.14-2.24]	0.006
Attending professional		
Obstetric nurse	1.00	
Obstetrician	2.82 [0.84-9.35]	0.092

PR - Prevalence ratio; CI - confidence interval

* Wald test. Deviance test (p value=0.897).

## DISCUSSION

This study estimated that more than half of the postpartum women interviewed experienced adequate or excellent well-being during childbirth. However, a significant proportion of participants reported distress, a condition that was associated with cesarean delivery and the lack of breastfeeding guidance. Other studies using the BMSP2 found higher prevalences of adequate or excellent well-being: 91.4%^(13)^; 87.2%^(14)^; 83.81%^(15)^; and 68%^(16)^. This finding, when compared with other studies, indicates the need for investment in improving care. The municipality where the study was conducted is a training hub for professionals in the region and has initiatives with the potential to contribute to this goal, such as the presence of two institutions with the BFHI title and residency programs in obstetrics and obstetric nursing.

Well-being during childbirth is closely related to the satisfaction of the woman in labor and involves multiple factors, such as clinical interventions and practices, the behaviors and attitudes of professionals, institutional infrastructure, and regional and global differences. Additionally, antenatal factors such as prior knowledge and preparation for childbirth during prenatal care are crucial for the quality of the experience. In general, professional interventions and practices that are not evidence-based for labor and delivery care, such as denying access to pain relief methods, disrespectful attitudes, poor communication, and inadequate prenatal practices, negatively impact satisfaction levels^(17,18)^. Maternal well-being also depends on a positive outcome, meaning a healthy newborn, the absence of complications, and the alignment of the birth process with the woman’s expectations^(19)^.

The “Continuous Family Participation” and “Quality of the Caregiver-Patient Relationship” domains of the BMSP2 showed the highest percentages of participants with a positive experience. Women who experience respectful care relationships, receive emotional support, and clear information report greater overall satisfaction with childbirth care^(18,20)^. Providing welcoming and respectful attention to opinions, values, and beliefs, as well as involving women in care decisions, contributes to a positive experience and increases perceived well-being^(13,21)^. The presence of a companion helps create a supportive environment, respecting privacy and providing emotional support during labor, which contributes to a positive experience and reduces the woman’s anxiety^(22)^. A national study on the implementation of humanization guidelines in care, involving 606 maternity hospitals, observed inadequacy in the presence of a companion in 8.4% of them. Only 56.9% of the institutions provided an adequate chair, and in only 75.6% was access to meals for the companion considered adequate^(23)^.

In contrast, the “Self-care and Comfort” and “Depersonalized Care” domains received the lowest evaluations according to the interviewees. “Self-care and Comfort” relates to nutrition during labor, the performance of exercises and activities for pain relief, and the woman’s choice of the most comfortable position during childbirth^(11)^. The “Depersonalized Care” domain assesses perceptions of poor physical conditions, psychological distress associated with the conduct of professionals, feelings of abandonment, and being subjected to procedures that do not align with a natural birth^(11)^.

The sample showed a predominance of good care practices, such as the presence of a companion, rooming-in, skin-to-skin contact with the newborn (NB) after birth, and support for breastfeeding. The presence of a companion is associated with better maternal and infant outcomes, adequate quality of care, and protection against verbal, psychological, and physical violence^(24,25)^. Breastfeeding within the first hour of life and rooming-in strengthens the mother-child bond and protects against early weaning^(26)^. The identified prevalence of breastfeeding within the first hour is higher than the national context, which is below 50%^(27)^. Early and continuous contact with the baby and the initiation of breastfeeding are factors associated with maternal satisfaction^(28)^.

A small percentage of postpartum women experienced practices that could negatively impact the childbirth process, such as the Kristeller maneuver, repetitive vaginal exams or exams performed by different people, and parallel conversations among professionals on unrelated topics. The Kristeller maneuver, a practice without scientific basis, should be abolished in childbirth care, although it is still performed, as indicated by prevalence rates of 37.3% and 23.1% found in the literature^(29,30)^. Practices not based on scientific evidence are considered obstetric violence and occur in about 60% of births in Brazil^(31)^. Vaginal exams, when performed frequently and without consent, also fall under this category of violence^(31,32)^. Other practices that interfere with the childbirth process, such as the unnecessary use of oxytocin, amniotomy, episiotomy, mandatory adoption of the lithotomy position, and cesareans without clinical indication, are also considered obstetric violence^(31-33)^.

According to Martins et al. (2021)^(18)^, satisfaction levels do not depend on the type of delivery but rather on the woman’s perception of the care provided during childbirth. However, other studies have found an association between cesarean sections and instrumental deliveries with lower levels of well-being^(4,34)^. After adjusting the multiple model, this study also found an association between distress and cesarean delivery. This result should be interpreted with caution, as it is necessary to distinguish between elective and non-elective cesareans, as the latter tend to provoke fewer positive feelings. It is also important to consider that the women investigated were mostly attended to in institutions within the Unified Health System (SUS in Portuguese) (89.4%), where performing a cesarean solely at the woman’s request is not possible in Minas Gerais.

When performed during labor, a cesarean section causes women to experience not only the pain, fear, tension, and anxiety associated with vaginal delivery but also the discomfort and pain of the post-surgical period. In addition to the disappointment of not having a vaginal birth, skin-to-skin contact with the baby in the delivery room-an important factor for well-being-is not performed in most cesarean deliveries, or when performed, does not maintain the same quality possible in vaginal births. This condition may explain the association found in this study.

Although the cesarean rate found is high, it is lower than the 2019 data for Minas Gerais, which reported 58.08%, and the national average of 56.3%, according to the SUS Department of Informatics^(4)^. Since 1985, the World Health Organization has considered that cesareans should account for 10% to 15% of total births. Despite this, and multiple global efforts to reduce it, cesarean rates have been increasing worldwide over the past three decades. Projections indicate that by 2030, cesarean births will be the predominant mode of delivery in Asia and Latin America, associated with the medicalization of childbirth, involving factors such as the values and beliefs of women and professionals^(3)^. These factors influence women’s preferences for the mode of delivery and, indirectly, the well-being and experience of childbirth.

In the adjusted model, the lack of breastfeeding guidance was associated with distress, indicating the importance of this domain for the birth experience. Robust scientific evidence demonstrates the reduction of maternal and infant morbidity and mortality with the implementation of actions that promote breastfeeding. In response, the BFHI was created to implement guidelines involving the training of professionals in technical and communication skills to promote and protect breastfeeding^(35)^. In the studied setting, two hospitals hold the BFHI title, which may explain the high prevalence of actions focused on breastfeeding. In contrast, a study^(36)^ evaluated 175 mother-baby pairs in the municipality of Montes Claros and found that only 45.7% of women received breastfeeding guidance while still in the hospital. Breastfeeding is a challenging event, as it requires skills, motivation, information, and support for mothers. The support received in the hospital is crucial for the mother to feel confident in facing the challenges that will arise at home and that may lead to the discontinuation of breastfeeding^(37)^. Educational interventions on breastfeeding can foster self-confidence and positively impact maternal well-being^(38)^.

Humanized care encompasses both the use of necessary and scientifically based interventions and the behavior of professionals focused on the needs of women. Providing a childbirth experience that is positive and satisfying is crucial for promoting maternal and child health. When the birth experience is distressing, psychological trauma can arise, negatively affecting women’s mental health throughout their lives, interfering with relationships with their children and spouses, and impacting future reproductive decisions^(39)^. Additionally, a traumatic hospital experience during childbirth can harm the newborn’s health by discouraging the pursuit of institutionalized care in future situations^(40)^.

The occurrence of distress during childbirth deprives women of the opportunity to experience joy, satisfaction, and empowerment, turning this moment into an unforgettable torment that cannot be replaced by a new experience. When professionals deny humanized care and cause distress during childbirth, it is a form of human rights violation that should be safeguarded by institutions^(41)^.

One strategy that has been increasingly adopted in recent years in the country is the inclusion of obstetric nurses in childbirth care. These professionals typically receive training within the humanized care paradigm and, by integrating into the team, can promote changes toward more empathetic and compassionate care, respecting the uniqueness of each woman in labor. Their continuous presence with the woman during labor alleviates anxiety and fear and reduces the number of interventions in care^(42)^.

### Study limitations

This study has several limitations. It was challenging to complete data collection from postpartum women during the COVID-19 pandemic in 2020. Due to the isolation imposed by the pandemic, it was necessary to use an online form to collect data, which limited the participation of some postpartum women. As a result, the minimum sample size of 208 participants, adjusted with an additional 10% for non-response and attrition, was not achieved. The interviews were conducted during a period proximate to labor, which is full of changes related to the postpartum period, potentially leading to recall bias. The non-probabilistic sampling prevents the generalization of the results. It was also not possible to distinguish between intrapartum and elective cesarean sections, which may impact the analysis of some items on the BMSP2.

### Contributions to Nursing, Health, or Public Policy

The findings are valuable for professionals and managers in discussing and improving the care provided to women during labor. There are few published studies in Brazil that use the BMSP2, and none have evaluated maternal well-being in women undergoing cesarean sections. This instrument is valid and reliable for assessing maternal well-being nationally and allows for comparisons across different settings, serving as a guide for designing public policies that promote the protagonism of women during childbirth.

## CONCLUSIONS

A significant proportion of maternal distress during childbirth was identified, associated with cesarean delivery and a lack of breastfeeding (AM) guidance. On the other hand, more than half of the postpartum women reported well-being during childbirth (adequate or excellent). The study also observed a predominance of good practices in obstetric and neonatal care. The results highlight the need for improvements in services, particularly in structural aspects, work processes, and professional conduct, with the aim of creating a care environment that promotes maternal satisfaction, normal delivery, and continuous support, in line with national and international recommendations.
