# Supplementary material for: Factors associated with maternal well-being during childbirth among postpartum women in Minas Gerais
Source: Rev Bras Enferm. 2024 Dec 16;77(6):e20230304. doi: 10.1590/0034-7167-2023-0304 (PMC11654529; doi:10.1590/0034-7167-2023-0304)
Supplement: Translation

# Translation: Fatores associados ao bem-estar materno em situação de parto de puérperas em Minas Gerais

**DOI:** 10.1590/0034-7167-2023-0304pt

**Authors:** Talyta Sâmara Batista Ferreira, Cinara Botelho Moutinho, Edmar Rocha Almeida, Ana Júlia Soares Oliveira, Carolina Amaral Oliveira Rodrigues, Clara de Cássia Versiani, Sibylle Emilie Vogt, Marise Fagundes Silveira

**Affiliations:** I Universidade Estadual de Montes Claros. Montes Claros, Minas Gerais, Brasil; II Hospital Santo Antônio. Taiobeiras, Minas Gerais, Brasil; III Secretaria Municipal de Saúde de Taiobeiras. Taiobeiras, Minas Gerais, Brasil

**Keywords:** Bem-estar Materno, Período Pós-Parto, Parto Obstétrico, Assistência ao Parto, Humanização da Assistência.

## Abstract

**Objetivos:**

analisar os fatores associados ao bem-estar materno em situação de parto de puérperas em Minas Gerais.

**Métodos:**

estudo transversal, aninhado a uma coorte, realizado com puérperas em um município mineiro. Foi adotada a escala Bem-Estar Materno em Situação de Parto 2. Estimaram-se as prevalências do bem-estar materno em situação de parto. A magnitude da associação entre mal-estar materno e práticas assistenciais foi estimada pela Razão de Prevalência (RP), utilizando-se a regressão de Poisson.

**Resultados:**

participaram 183 puérperas com idade entre 15 e 46 anos, sendo que 26,2%, 27,9% e 45,9% relataram, respectivamente, ótimo, adequado e mal-estar na assistência ao parto. O mal-estar materno foi mais prevalente entre puérperas que passaram por parto cesárea (RP = 1,60) e que não receberam informações sobre amamentação (RP = 1,59).

**Conclusões:**

observou-se elevada prevalência de mal-estar no parto, associada à realização de cesáreas e à falta de informações sobre amamentação.

## INTRODUÇÃO

Desde a década de 1990, foram registrados avanços importantes nas políticas públicas de saúde materno-infantil no Brasil, com foco na humanização da assistência, visando qualificar a atenção ao pré-natal, parto e nascimento, garantir acesso e acolhimento às gestantes e reduzir a mortalidade infantil e materna. O modelo assistencial almejado tem como pilares o respeito às demandas das mulheres e à fisiologia do trabalho de parto e parto, a oferta de informações objetivas, relações interpessoais entre parturiente e profissional empáticas e não autoritárias, a inclusão das mulheres na tomada de decisões e o trabalho em equipe com reconhecimento e valorização de enfermeiros obstétricos e obstetrizes, além do uso de práticas e condutas assistenciais baseadas em evidências^(1,2)^.

Ainda assim, condutas inapropriadas são recorrentes na realidade assistencial obstétrica e neonatal. Mulheres e recém-nascidos são submetidos a intervenções nem sempre necessárias, como o uso rotineiro de ocitocina, episiotomia, cesariana, aspiração nasofaríngea do neonato, dentre outras. Além disso, o relato de desrespeito por parte dos profissionais nas interações e relações com as parturientes é frequente^(1,2)^.

Dados publicados pela World Health Organization (WHO) mostram que, entre 2010 e 2018, os nascimentos por cesariana corresponderam a 21,1% no mundo, 42,8% na América Latina e no Caribe, e 55,7% no Brasil, que ocupa o segundo lugar global^(3)^. No ano de 2019, a proporção de cesáreas representou 58,08% em Minas Gerais e 49,71% no município de Montes Claros, cenário do estudo^(4)^. A cidade possui três hospitais que oferecem serviços de assistência ao parto, predominantemente com financiamento público, sendo dois deles cenários de formação de médicos obstetras e um de enfermeiros obstetras.

Para além de um parto seguro, com condutas baseadas em evidências científicas, é preciso garantir uma experiência positiva para as parturientes e suas famílias^(1,5)^. O bem-estar na situação do parto e a satisfação com a vivência referem-se à percepção, pela mulher, das atitudes, comportamentos e condutas assistenciais assumidas pelos profissionais. A satisfação com a vivência do parto também é influenciada pelo atendimento das expectativas, nível de informação das mulheres e pelo resultado do parto, isto é, se o parto resultou em um recém-nascido saudável e não houve complicações maternas ou neonatais^(6)^. Sentir-se acolhida e compreendida em suas demandas pelos profissionais, com respeito à sua privacidade e a possibilidade de exercer a autonomia no processo assistencial, junto com uma estrutura de qualidade, são as demandas das mulheres e geram bem-estar e satisfação^(7)^.

A gestação e o parto são momentos singulares, marcados por fortes emoções, que colocam as mulheres em situações de extrema vulnerabilidade. Vivências negativas, abusos e violência obstétrica sofridos durante a assistência estão associados a transtornos depressivos, influenciando de forma negativa a qualidade de vida das mulheres e sua capacidade de cuidar de si e do recém-nascido^(8,9)^. As experiências relacionadas ao parto são marcadas pela subjetividade, entretanto, podem ser mensuradas objetivamente, o que tem sido objeto de inúmeras propostas de avaliação^(10)^. Um dos instrumentos validados para a realidade cultural brasileira^(11)^ é a Escala de Bem-Estar em Situação de Parto 2 (BMSP2)^(11)^, que tem sido adotada em estudos nacionais para estimar a prevalência do bem-estar no parto^(12,13)^.

É importante avaliar o bem-estar materno como um marcador de qualidade assistencial. Poucos trabalhos investigam esse desfecho do cuidado, predominando estudos qualitativos. Além disso, a maioria das pesquisas quantitativas não utiliza instrumentos validados para a avaliação do bem-estar durante e após o parto^(10)^.

## OBJETIVOS

Analisar os fatores associados ao bem-estar materno em situação de parto de puérperas em Minas Gerais.

## MÉTODOS

### Aspectos éticos

O estudo foi conduzido de acordo com as diretrizes éticas nacionais e internacionais e aprovado pelo Comitê de Ética em Pesquisa da Universidade Estadual de Montes Claros, cujas aprovações estão anexadas à presente submissão. O Consentimento Livre e Esclarecido foi obtido de todas as participantes com idade acima de 18 anos, por meio de assinatura. As participantes com idade igual ou inferior a 18 anos, bem como seus responsáveis, assinaram, respectivamente, o Termo de Assentimento Livre e Esclarecido (TALE) e o Termo de Consentimento Livre e Esclarecido (TCLE).

### Desenho, local do estudo e período

Estudo epidemiológico transversal e analítico, aninhado ao “Estudo ALGE - Avaliação das condições de saúde das gestantes de Montes Claros-MG: estudo longitudinal”, realizado na zona urbana do município de Montes Claros, MG, Brasil, entre 2018 e 2020. O Estudo ALGE teve como objetivo conhecer as condições de saúde das gestantes, puérperas e crianças assistidas nos serviços da Estratégia de Saúde da Família (ESF) de Montes Claros.

### População ou amostra, critérios de inclusão e exclusão

O “Estudo ALGE” foi realizado em três momentos. A população do 1º momento (baseline) foi constituída por todas as gestantes (N=1661) cadastradas na ESF entre 2018 e 2019. Destas, as que estavam no 1º trimestre gestacional (N=448) foram convidadas a participar do 2º momento, quando se encontravam no 3º trimestre de gravidez, e do 3º momento, quando puérperas, 40 a 70 dias após o parto.

O tamanho da amostra do baseline (1º momento) foi estabelecido visando estimar parâmetros populacionais com prevalência de 50%, intervalo de confiança de 95% (nível de precisão de 2,0%). Foi feita uma correção para população finita (N=1.661 gestantes) e estabelecido um acréscimo de 20% para compensar possíveis não respostas e perdas. Os cálculos indicaram a necessidade de participação de, no mínimo, 1.180 gestantes.

O presente estudo refere-se ao 3º momento dessa coorte, cuja população foi composta por 448 puérperas, cadastradas em equipes da ESF da zona urbana de Montes Claros, que estavam no 1º trimestre gestacional no 1º momento do “Estudo ALGE”. Foram elegíveis as puérperas cujo parto ocorreu durante o período do estudo. Foram excluídas as puérperas que tiveram gravidez gemelar e aquelas que não receberam assistência imediata ao parto (parto domiciliar).

Para o cálculo do tamanho amostral do 3º momento do “Estudo ALGE”, adotou-se a fórmula para estudos transversais, com correção para população finita (N=448), utilizando os seguintes parâmetros: proporção populacional estimada de 50%, margem de erro de 5%, nível de confiança de 95% e acréscimo de 10% para a taxa de não resposta e perdas, resultando em 229 puérperas.

Não houve seleção aleatória da amostra; todas as gestantes cadastradas na ESF entre 2018 e 2019 foram convidadas a participar do estudo.

### Protocolo do estudo

Em 2019, os dados foram coletados por meio de entrevistas presenciais, realizadas nos domicílios das mulheres em horário previamente agendado. Em 2020, devido à pandemia da COVID-19, os dados foram coletados por meio de um formulário *online* (*Google Forms*), cujo *link* foi enviado para o *e-mail* ou *WhatsApp* das puérperas. A equipe de entrevistadores, composta por profissionais da saúde e acadêmicos vinculados à iniciação científica, recebeu treinamento para padronizar o procedimento.

Foram analisadas as seguintes variáveis: (1) características sociodemográficas das puérperas (idade, escolaridade, estado civil e fonte de pagamento para o parto); (2) variáveis assistenciais do parto (profissional assistente do parto, tipo de parto, presença de acompanhante, posição durante o parto, manobra de Kristeller, toques repetitivos ou realizados por pessoas diferentes, conversas paralelas entre os profissionais sobre outros assuntos durante o parto, destino do recém-nascido após o parto, contato pele a pele com o recém-nascido, aleitamento materno na primeira hora, recebimento de orientações sobre o aleitamento materno e apoio para o aleitamento materno); e (4) bem-estar materno no parto.

Para avaliar o bem-estar materno no parto, foi utilizada a escala Bem-Estar Materno em Situação de Parto 2 (BMSP2), adaptada culturalmente e validada para o português do Brasil^(11)^. Este instrumento possui 47 itens com opções de resposta em escala do tipo Likert, variando de um (discordo totalmente) a cinco (concordo totalmente). Os itens são distribuídos em sete domínios: qualidade do relacionamento durante o cuidado (13 itens), autocuidado e conforto (9 itens), condições que propiciam o contato entre mãe e filho (4 itens), cuidado despersonalizado (6 itens), participação familiar contínua (4 itens), cuidado oportuno e respeitoso (6 itens) e ambiente físico confortável (5 itens). O escore total da escala estabelece três níveis de bem-estar materno: ótimo (pontuação > 200), adequado (pontuação > 183 e < 200) e mal-estar (pontuação < 183)^(11)^.

### Análise dos resultados e estatística

As variáveis categóricas foram descritas por meio de suas distribuições de frequências absolutas e relativas. A pontuação dos domínios da escala BMSP2 foi obtida pela soma das respostas aos respectivos itens, e a pontuação total da escala, pela soma das respostas dadas a todos os itens. Ressalta-se que a pontuação dos itens 5, 32, 33, 35 e 43, referentes ao domínio “cuidado despersonalizado”, foi invertida antes da somatória.

Foram calculadas as medidas descritivas (média, desvio padrão, valores máximo e mínimo) da pontuação total e dos sete domínios da escala, bem como os valores dos pontos de corte que corresponderam a 77,8% do valor máximo possível de cada domínio e do escore total^(12)^. Esse percentil (77,8%) foi definido em um estudo sobre as propriedades psicométricas da escala BMSP2^(12)^. Também foram estimadas as proporções de mulheres que apresentaram pontuação nos domínios e no escore total da escala superior aos respectivos pontos de corte, demonstrando experiência positiva na assistência ao parto^(12)^. Foram estimadas as prevalências, com intervalos de 95% de confiança, para as três categorias do bem-estar materno no parto (ótimo, adequado e mal-estar), e construídos um histograma e um gráfico de colunas para a apresentação dos escores obtidos.

Para avaliar a associação entre bem-estar materno no parto (variável dependente) e as características sociodemográficas, idade gestacional e variáveis relativas à assistência ao parto (variáveis independentes), as categorias da variável dependente “ótimo” e “adequado” foram agrupadas, pois representam resultados positivos. Foi conduzida uma análise bivariada por meio do teste Qui-quadrado ou Exato de Fisher. As variáveis independentes que apresentaram associação significativa com o bem-estar materno no parto, ao nível de 0,25, foram selecionadas para a análise múltipla.

Na análise múltipla, foi adotado o modelo de regressão de Poisson, com variância robusta. Foram estimadas as razões de prevalência (RP), com intervalos de 95% de confiança. O método *backward* (para trás) foi utilizado para a entrada das variáveis no modelo múltiplo. Para ajuste do modelo, permaneceram no modelo final as variáveis que apresentaram valor-p ≤0,10. O nível de significância de 0,05 foi adotado para considerar que a associação foi significativa com a variável dependente. O Teste de *Deviance* foi utilizado para avaliar a qualidade de ajuste do modelo final. Os dados foram analisados utilizando o software *IBM SPSS Statistics* versão 23.0 para *Windows* ^®^.

## RESULTADOS

Participaram do estudo 183 puérperas, cuja idade variou de 15 a 46 anos, com média de 26,3 anos. A média do tempo pós-parto em que foi realizada a entrevista foi de 59,6 dias (desvio-padrão = 12,4 dias). A maioria (49,2%) estava na faixa etária entre 20 e 29 anos, e mais de um terço (67,6%) possuía o ensino médio incompleto ou completo. As demais características sociodemográficas e relacionadas à assistência ao parto das puérperas estão descritas na Tabela 1.

**Tabela 1:** Distribuição das puérperas segundo variáveis sociodemográficas e de assistência ao parto, Montes Claros, Minas Gerais, Brasil, 2019-2020

Variável	n^*^	%
Variáveis sociodemográficas		
Faixa etária (anos)		
15 a 19 anos	29	15,8
20 a 29 anos	90	49,2
30 anos ou mais	64	35,0
Escolaridade		
Fundamental incompleto ou completo	23	12,6
Médio incompleto ou completo	123	67,6
Superior incompleto ou completo	36	19,8
Situação Conjugal		
Casada ou União consensual	139	75,9
Solteira/ Separada/Desquitada/Divorciada/Viúva	44	24,1
Fonte de pagamento para o parto		
Público (SUS)	164	89,6
Privado (convênio ou particular)	19	10,4
Variáveis de assistência ao parto		
Tipo de parto		
Normal	102	55,7
Cesárea	81	44,3
Profissional assistente		
Médico(a) obstetra	164	**92,1**
Enfermeiro(a) obstetra	14	**7,9**
Presença de acompanhante	174	95,1
Posição horizontalizada durante o parto^1^	88	86,3
Manobra de Kristeller	10	5,5
Toques vaginais repetitivos	30	16,4
Toques vaginais realizados por diferentes pessoas	19	10,4
Conversas paralelas entre os profissionais	31	16,9
RN ficou com a mãe depois do parto	156	85,2
Contato pele a pele com o RN após o nascimento	166	90,7
AM na primeira hora	142	77,6
Recebeu orientações sobre o AM	162	88,5
Recebeu apoio para AM	150	88,5
Total	183	100,0

* *Os totais variam devido aos dados perdidos; 1Amostra de mulheres que tiveram parto normal; RN - recém-nascido; AM - aleitamento materno.*

Quanto ao bem-estar materno em situação de parto, a Tabela 2 apresenta os valores obtidos para os domínios e para o escore total da BMSP2. Quanto maior o valor alcançado, mais positiva será a avaliação do bem-estar. Os valores do escore total da escala variaram de 116,0 a 235,0, com média de 184,7 e percentis 25 e 75, respectivamente, iguais a 168,0 e 201,0 (Figura 1A). Com exceção dos domínios “Autocuidado e conforto” e “Cuidado despersonalizado”, todos os demais apresentaram valores médios acima do ponto de corte, que correspondeu a 77,8% do valor total possível. Quanto ao escore total, observa-se que 54,1% das mulheres experimentaram positivamente (adequado ou ótimo) a assistência ao parto, isto é, apresentaram valores superiores ao ponto de corte. A proporção das três categorias de bem-estar, de acordo com a escala BMSP2, está apresentada na Figura 1B.

**Tabela 2:** Medidas descritivas da Escala de Bem-Estar Materno em Situação de Parto 2 e classificação das puérperas de acordo com os escores da escala, Montes Claros, Minas Gerais, Brasil, 2019-2020

Domínios da escala BMSP 2	Nº de itens	Intervalo possível	Intervalo obtido	Média (d.p)	Ponto de corte^*^ de 77,8%	% acima do ponto de corte^**^
I- Qualidade do relacionamento durante o cuidado	13	13 - 65	28 - 65	54,4 (8,4)	50,6	80,3
II- Autocuidado e conforto	9	9 - 45	9 - 45	28,4 (8,8)	35,0	21,3
III- Condições que propiciam o contato entre mãe e filho	4	4 - 20	4 - 20	16,5 (3,0)	15,6	74,9
IV- Cuidado despersonalizado	6	6 - 30	8 - 30	22,5 (4,1)	23,4	45,9
V- Participação familiar contínua	4	4 - 20	11 - 20	17,3 (2,1)	15,6	85,2
VI- Cuidado oportuno e respeitoso	6	6 - 30	13 - 30	24,6 (3,5)	23,4	67,8
VII- Ambiente físico confortável	5	5 - 25	11 - 25	20,1 (3,0)	19,5	75,4
Total	47	47 - 235	116 - 235	184,7 (25,0)	183,0	54,1
**Classificação do BMSP 2**	**Pontuação**			**n**	**% [IC95%]**	
Mal-estar	< 183			84	45,9 [38,5 - 52,9]	
Adequado	183 ≥ x ≥ 200			51	27,9 [22,0 - 35,9]	
Ótimo	> 200			48	26,2 [20,5 - 33,2]	
Total	-			183	100,0	

BMSP - Bem-estar materno em situação de parto; d.p: desvio-padrão;

* Ponto de corte - 77,8% do valor máximo possível;

** Percentual de mulheres com escores acima do ponto de corte; IC - intervalo de confiança.

**Figura 1:** Distribuição do escore total da escala de Bem-Estar Materno em Situação de Parto (Figura 1A) e classificação das puérperas de acordo com a pontuação da escala (Figura 1B), Montes Claros, Minas Gerais, Brasil, 2019-2020

As seguintes variáveis relativas à assistência ao parto apresentaram associação significativa, ao nível de 0,25, com o bem-estar materno no parto: profissional assistente do parto, tipo de parto, conversas paralelas entre os profissionais sobre outros assuntos durante o parto e recebimento de orientações sobre o aleitamento materno. Essas variáveis foram selecionadas para a análise múltipla (Tabela 3).

**Tabela 3:** Associação entre o Bem-estar Materno em Situação de Parto e as variáveis sociodemográficas e de assistência ao parto, Montes Claros, Minas Gerais, Brasil, 2019-2020

Variáveis	Classificação da escala BMSP2		Valor de *p* ^*^
	Adequado/Ótimo	Mal-estar	
	n (%)	n (%)	
Variáveis sociodemográficas			
Idade materna			0,956
15 a 19 anos	16 (56,7)	13(43,3)	
20 a 29 anos	49 (53,9)	41(46,1)	
30 anos ou mais	34 (53,4)	30 (46,1)	
Escolaridade			0,444
Fundamental incompleto ou completo	10 (42,9)	13 (57,1)	
Médio incompleto ou completo	66 (53,7)	57 (46,3)	
Superior incompleto ou completo	22 (60,6)	14 (46,3)	
Situação Conjugal			0,446
Casada/União consensual	73 (52,5)	66 (47,5)	
Solteira/Separada/Divorciada/Viúva	26 (59,1)	18 (40,9)	
Fonte de pagamento para o parto			0,892
Público (SUS)	89 (54,3)	75 (45,7)	
Privado (convênio ou particular)	10 (52,6)	9 (47,4)	
Variáveis de assistência ao parto			
Profissional assistente			0,013
Médico(a) obstetra	84 (51,2)	80 (48,8)	
Enfermeiro(a) obstetra	12 (85,7)	2 (14,3)	
Tipo de parto			**<0,001**
Normal	68 (66,7)	34 (33,3)	
Cesárea	31 (38,3)	50 (61,7)	
Presença de acompanhante	95 (54,6)	79 (45,4)	0,551
Posição horizontalizada do parto^1^	59 (65,9)	29 (34,1)	0,861
Manobra de Kristeller	7 (70,0)	3 (30,0)	0,299
Toques vaginais repetitivos	14 (46,7)	16 (53,3)	0,372
Toques vaginais realizados por diferentes pessoas	9 (47,4)	10 (52,6)	0,534
Conversas paralelas entre os profissionais	12 (38,7)	19 (61,3)	0,059
RN ficou com a mãe depois do parto	86 (55,1)	70 (44,9)	0,401
Contato pele a pele com o RN após o nascimento	91 (54,8)	75 (45,2)	0,541
AM na primeira hora	78 (54,9)	64 (45,1)	0,675
Recebeu orientações sobre o AM	93 (57,4)	69 (42,6)	0,013
Recebeu apoio para AM	83 (55,3)	67 (44,7)	0,475
Total	99 (54,1)	84 (45,9)	

BEMSP - bem-estar materno em situação de parto;

* Teste Qui-quadrado; ^1^Amostra de mulheres que tiveram parto normal; RN - Recém-nascido; AM - Aleitamento materno.

A Tabela 4 apresenta os resultados da análise múltipla. Observa-se que a prevalência de mal-estar no parto foi 60,0% maior entre as mulheres cujo parto foi cesáreo comparadas com aquelas que tiveram parto normal (RP=1,60). Entre as mulheres que não receberam orientações sobre o aleitamento materno, a prevalência de mal-estar no parto foi 59,0% maior que aquelas que receberam as orientações (RP=1,59). As demais variáveis analisadas não apresentaram associação significativa com o mal-estar no parto após ajuste do modelo. O modelo final apresentou qualidade de ajuste (teste de *Deviance* valor-p = 0,897).

**Tabela 4:** Modelo de regressão de Poisson ajustado das variáveis relativas à assistência ao parto associadas ao Mal-estar no parto, Montes Claros, Minas Gerais, Brasil, 2019-2020

Variáveis	RP [IC95%]	Valor de *p* ^*^
Tipo de parto		
Normal	1,00	
Cesárea	1,60 [1,17-2,21]	0,004
Recebeu orientações sobre o aleitamento materno		
Sim	1,00	
Não	1,59 [1,14-2,24]	0,006
Profissional assistente		
Enfermeiro(a) obstetra	1,00	
Médico(a) obstetra	2,82 [0,84-9,35]	0,092

RP - Razão de prevalência; IC - intervalo de confiança

* Teste de wald. Teste deviance (valor de p=0,897).

## DISCUSSÃO

Este estudo estimou que mais da metade das puérperas entrevistadas experimentou bem-estar adequado ou ótimo no parto. No entanto, uma proporção importante de participantes relatou mal-estar, um desfecho que esteve associado ao parto cesáreo e à falta de orientações sobre o aleitamento materno (AM). Outros estudos que usaram a BMSP2 encontraram prevalências mais elevadas de bem-estar adequado ou ótimo: 91,4%^(13)^; 87,2%^(14)^; 83,81%^(15)^; e 68%^(16)^. Esse achado, quando comparado com outros estudos, indica a necessidade de investimentos na melhoria do cuidado. O município onde o estudo foi conduzido é um polo formador de profissionais para a região e possui iniciativas com potencial para contribuir com esse objetivo, como a presença de duas instituições com o título da Iniciativa Hospital Amigo da Criança (IHAC) e programas de residência médica em obstetrícia e em enfermagem obstétrica.

O bem-estar no parto está estreitamente relacionado com a satisfação da parturiente e envolve múltiplos fatores, como: as intervenções e condutas clínicas; os comportamentos e atitudes dos profissionais; a infraestrutura institucional; e as diferenças regionais e globais. Além disso, aspectos antenatais, como conhecimento prévio e preparação para o parto durante o pré-natal, são decisivos para a qualidade da vivência. Em geral, intervenções e práticas profissionais não fundamentadas em evidências científicas para a assistência ao trabalho de parto e parto, como negar o acesso a métodos de alívio da dor, atitudes desrespeitosas, comunicação deficiente e condutas inadequadas no pré-natal, interferem negativamente no nível de satisfação^(17,18)^. O bem-estar materno também depende de um desfecho positivo, ou seja, um recém-nascido saudável, a ausência de complicações e a adequação do processo parturitivo às expectativas da mulher^(19)^.

Os domínios “Participação familiar contínua” e “Qualidade do relacionamento durante o cuidado” da BMSP2 apresentaram as maiores porcentagens de participantes com experiência positiva. Mulheres que vivenciam relações respeitosas de cuidado, recebem apoio emocional e informações claras relatam maior satisfação global com a assistência ao parto^(18,20)^. O acolhimento e o respeito às opiniões, valores e crenças, bem como o envolvimento nas decisões assistenciais, contribuem para uma vivência positiva e aumentam o bem-estar percebido^(13,21)^. A presença de um acompanhante facilita a criação de um ambiente acolhedor, com respeito à privacidade e apoio emocional durante o trabalho de parto, contribuindo para uma experiência positiva e redução da ansiedade da parturiente^(22)^. Um estudo nacional sobre a implementação das diretrizes de humanização na assistência, envolvendo 606 maternidades, observou inadequação para a presença do acompanhante em 8,4% delas. Apenas 56,9% das instituições dispunham de uma poltrona adequada, e somente em 75,6% o acesso às refeições para o acompanhante foi classificado como adequado^(23)^.

Em contraste, os domínios “Autocuidado e conforto” e “Cuidado despersonalizado” registraram as piores avaliações segundo as entrevistadas. O “Autocuidado e conforto” relaciona-se com a alimentação durante o trabalho de parto, a realização de exercícios e atividades para alívio da dor, e a escolha da parturiente pela posição mais confortável^(11)^. O domínio “Cuidado despersonalizado” afere as percepções sobre as más condições do ambiente físico, o mal-estar psicológico associado à conduta dos profissionais, o sentimento de abandono e a submissão a procedimentos que não correspondem a um parto natural^(11)^.

Observou-se um predomínio na amostra de boas práticas de cuidado, como a presença de acompanhante, o alojamento conjunto, o contato pele a pele com o recém-nascido (RN) após o nascimento e o apoio para o aleitamento materno (AM). A presença do acompanhante está associada a melhores desfechos materno-infantis, à qualidade adequada da assistência, e promove proteção contra a ocorrência de violência verbal, psicológica e física^(24,25)^. A amamentação na primeira hora de vida e o alojamento conjunto fortalecem o vínculo mãe-filho e protegem contra o desmame precoce^(26)^. A prevalência identificada do AM na primeira hora de vida é maior que o estimado para o contexto nacional, que é inferior a 50%^(27)^. O contato precoce e contínuo com o bebê e o início da amamentação são fatores associados à satisfação materna^(28)^.

Uma pequena porcentagem das puérperas experimentou práticas que podem interferir negativamente na vivência do processo parturitivo, como a manobra de Kristeller, toques vaginais repetitivos ou realizados por diferentes pessoas, e conversas paralelas entre os profissionais sobre outros assuntos. A manobra de Kristeller, prática sem fundamento científico, deve ser abolida na assistência ao parto, embora ainda seja realizada, como indicado por prevalências de 37,3% e 23,1% encontradas na literatura^(29,30)^. Condutas não fundamentadas em evidências científicas são consideradas violência obstétrica e ocorrem em cerca de 60% dos partos no Brasil^(31)^. O exame vaginal, quando realizado de forma frequente e sem consentimento, também se enquadra nesse tipo de violência^(31,32)^. Outras condutas que interferem no processo de parturição, como o uso desnecessário de ocitocina, amniotomia, episiotomia, adoção obrigatória da posição litotômica, bem como cesáreas sem indicação clínica, também são consideradas violência obstétrica^(31-33)^.

Segundo Martins et al. (2021)^(18)^, o nível de satisfação não depende do tipo de parto, mas sim da percepção sobre os cuidados dispensados à mulher durante a parturição. No entanto, outros estudos encontraram associação entre cesarianas e partos instrumentalizados e níveis menores de bem-estar^(4,34)^. Após ajuste do modelo múltiplo, este estudo também evidenciou uma associação entre o mal-estar e o parto cesáreo. Esse resultado deve ser interpretado com cautela, pois é necessário distinguir entre cesarianas eletivas e não programadas, visto que estas últimas tendem a provocar menos sentimentos positivos. Deve-se considerar que as parturientes investigadas foram majoritariamente atendidas em instituições inseridas no Sistema Único de Saúde (SUS) (89,4%), onde a realização da cesárea apenas pelo desejo da mulher não é possível em Minas Gerais.

Quando realizada intraparto, a cesariana faz com que as mulheres experimentem não apenas as dores, os medos, as tensões e as ansiedades próprias do parto vaginal, mas também os desconfortos e dores do pós-cirúrgico. Além da quebra da expectativa por um parto vaginal, o contato pele a pele com o bebê ainda na sala de parto, um fator importante para o bem-estar, não é realizado na maioria dos partos cesáreos, ou quando realizado, não mantém a mesma qualidade possível no parto vaginal. Essa condição pode justificar a associação encontrada neste estudo.

Embora a taxa de cesárea encontrada seja de prevalência elevada, ela é inferior aos dados de 2019 em Minas Gerais, que apontam 58,08%, e à média nacional de 56,3%, segundo o Departamento de Informática do SUS^(4)^. Desde 1985, a Organização Mundial de Saúde considera que as cesáreas devem corresponder de 10% a 15% do total de partos. Apesar disso, e dos múltiplos esforços globais para sua redução, as taxas de cesariana no mundo vêm aumentando nas últimas três décadas. Projeções indicam que, até 2030, o nascimento via cesariana será o modo predominante na Ásia e América Latina, sendo associado à medicalização do parto, envolvendo fatores como valores e crenças das mulheres e dos profissionais^(3)^. Esses fatores influenciam as preferências das mulheres pela via de parto e, indiretamente, o bem-estar e a vivência das parturientes.

No modelo ajustado, a falta de orientações sobre o aleitamento materno (AM) esteve associada ao mal-estar, indicando a importância desse domínio para a experiência de nascimento. Evidências científicas robustas demonstram a redução da morbimortalidade materna e infantil com a implementação de ações que favoreçam a amamentação. Diante disso, foi criada a Iniciativa Hospital Amigo da Criança (IHAC) com a finalidade de implementar diretrizes que envolvem o treinamento dos profissionais em habilidades técnicas e de comunicação para a promoção e proteção do aleitamento materno^(35)^. No cenário estudado, dois hospitais possuem o título da IHAC, o que pode explicar a elevada prevalência de ações voltadas para o AM. Em contraste, um estudo^(36)^ avaliou 175 binômios mãe-bebê no município de Montes Claros e encontrou que apenas 45,7% das mulheres receberam orientações sobre AM ainda na maternidade. O AM é um evento desafiador, pois requer habilidades, motivação, informação e apoio para as mães. O suporte recebido ainda na maternidade é crucial para que a mãe se sinta segura no enfrentamento das dificuldades que surgirão em domicílio e que podem levar à interrupção da amamentação^(37)^. Intervenções educativas sobre AM podem favorecer a autoconfiança e impactar positivamente o bem-estar materno^(38)^.

Uma assistência humanizada engloba tanto o uso de intervenções necessárias e cientificamente fundamentadas quanto o comportamento dos profissionais centrado nas demandas das mulheres. Proporcionar um trabalho de parto vivenciado de forma positiva e com satisfação é crucial para a promoção da saúde materna e infantil. Quando a experiência de parto é atormentadora, podem surgir traumas psicológicos que afetam negativamente a saúde mental das mulheres ao longo da vida, interferindo nas relações com a prole e cônjuges, além de impactar as decisões reprodutivas posteriores^(39)^. Além disso, uma experiência hospitalar traumática durante o parto pode causar danos à saúde do recém-nascido, ao desestimular a busca por assistência institucionalizada em ocasiões futuras^(40)^.

A ocorrência de mal-estar no parto priva as mulheres da oportunidade de vivenciar a alegria, a satisfação e o empoderamento, transformando esse momento em um tormento inesquecível, que não pode ser substituído por uma nova vivência. A atuação dos profissionais ao negar uma assistência humanizada e causar mal-estar no parto é uma forma de violação dos direitos humanos, que devem ser assegurados pelas instituições^(41)^.

Uma estratégia adotada de forma crescente nos últimos anos no país é a inclusão da assistência ao parto prestada por enfermeiros obstétricos. Esses profissionais, em geral, recebem formação dentro do paradigma da assistência humanizada e, ao integrar a equipe, podem promover mudanças rumo a um cuidado mais empático e solidário, respeitando a singularidade de cada parturiente. Sua presença contínua junto à parturiente alivia a ansiedade e o medo, além de reduzir as intervenções na assistência^(42)^.

### Limitações do estudo

O presente estudo apresenta limitações. Foi um desafio concluir a coleta dos dados das puérperas durante a pandemia da COVID-19 em 2020. Devido ao isolamento imposto pela pandemia, foi necessário adotar um formulário *online* para a obtenção dos dados, o que comprometeu a participação de uma parcela das puérperas. Assim, o tamanho mínimo amostral de 208 gestantes, descontado o acréscimo de 10% para a taxa de não-respostas e perdas, não foi alcançado. As entrevistas ocorreram em um período prospectivo ao trabalho de parto, que é repleto de mudanças relacionadas ao puerpério, o que pode ter causado viés de memória. A amostragem não probabilística impede a generalização dos resultados. Não foi possível distinguir as cesáreas intraparto das eletivas, o que pode impactar a análise de alguns itens da BMSP2.

### Contribuições para a área da enfermagem, saúde ou política pública

Os achados são úteis para profissionais e gestores na discussão e aprimoramento dos cuidados dispensados às mulheres no trabalho de parto. Existem poucos estudos publicados no Brasil que utilizam a BMSP2, e nenhum deles avaliou o bem-estar materno em mulheres submetidas a cesariana. Esse instrumento é válido e confiável para avaliar o bem-estar materno em território nacional e permite comparar diferentes cenários, servindo como norteador para o desenho de políticas públicas que promovam o protagonismo da parturiente.

## CONCLUSÕES

Identificou-se uma proporção significativa de mal-estar materno no parto, associada ao parto cesáreo e à falta de orientações sobre o aleitamento materno (AM). Por outro lado, mais da metade das puérperas relataram bem-estar durante o parto (adequado ou ótimo). Observou-se também um predomínio de boas práticas no cuidado obstétrico e neonatal. Os resultados destacam a necessidade de melhorias nos serviços em aspectos estruturais, nos processos de trabalho e nas condutas profissionais, com o objetivo de implementar um ambiente assistencial que promova a satisfação da parturiente, o parto normal e o apoio contínuo, conforme recomendações nacionais e internacionais.
